# Spatial–temporal evolution of ESV and its response to land use change in the Yellow River Basin, China

**DOI:** 10.1038/s41598-022-17464-w

**Published:** 2022-07-30

**Authors:** Jie Yang, Baopeng Xie, Degang Zhang

**Affiliations:** 1 College of Pratacultural Science, Gansu Agricultural University, Lanzhou, 730070 China; 2 College of Management, Gansu Agricultural University, Lanzhou, 730070 China

**Keywords:** Ecology, Ecosystem ecology, Ecosystem services

## Abstract

The value of ecosystem services, as well as their temporal and spatial characteristics, can be used to help areas develop focused and localized sustainable ecological management plans. Thus, this study conducted in the Yellow River Basin (YRB) of China, analyzed the ecosystem service value (ESV) and its spatial–temporal variation characteristics. This study used the equivalent factor and geospatial exploration methods, introduced the elasticity coefficient, and explored the response of ESV change to land-use change, based on the land use cover data from 1990 to 2020. The results showed that from 1990 to 2020, YRB ecosystem service value showed an overall increasing trend, mainly because the ecological construction project increased forest and grasslands in this region. In the past 30 years, spatial characteristics of ESV in YRB was relatively stable. The high-value areas were mainly distributed in the upper Yellow River Basin, while the low-value areas were mainly distributed in the lower Yellow River Basin, as the cold and hot spots were reduced. The ESV barycenter coordinates showed the direction of the transfer trajectory, which is first to southwest, northeast, and then to southwest. From 2000 to 2010, YRB land-use change had greater impact on ESV. Since 2010, the disturbance of ecosystem services by land-use change has decreased. Consequently, the elastic index of the upstream and Loess Plateau regions were significantly higher than that of other regions, and the impact of land-use change on ecosystem services was more obvious, due to improved large-scale ecological construction projects implementation. Conclusively, this study recommends the use of comprehensive spatial–temporal assessment of ESV for sustainable development and ecological protection in the YRB.

## Introduction

The notion of nature's services, also known as ecosystem services, was created to highlight the benefits that ecosystems provide to society and to raise awareness about the importance of biodiversity conservation^1^. Ecosystem services are related to human’s well-being and are the foundation for human survival and socio-economic development. Since the release of the United Nation’s Millennium Ecosystem Assessment report, ecosystem services and assessment research has received considerable global attention^2^. Understanding external and internal spatial–temporal evolution and impact of ecosystem service mechanisms are of great importance for identifying regional ecosystem service problems, maintaining regional ecological balance, and promoting regional sustainable development. Among them, ecosystem service value in the form of currency was widely used by decision-makers, due to its ease of understanding, effective spatial planning, and ecological regulation and restoration.The ESV is estimated by using ecological parameter model based on energy or the equivalent method based on value per unit area^3–7^. In 1997, Costanza proposed an equivalent factor of global ecosystem value to measure the value of global ecosystem services, and it has been widely used in the assessment of ESV around the world^8^. On this foundation, Xie et al. established an ESV assessment system for different terrestrial ecosystems in China^9^. Many scholars make adjustments and improvements based on the ecosystem and socioeconomic development status of the study area^10,11^, allowing for a more accurate assessment of regional ecosystem status. The value of ecosystem services is the foundation for describing the spatial–temporal evolution of ecosystem services, and accurate accounting is critical to scientific management of ecosystems and the realization of sustainable development.

At present, research on ESV mainly focuses on three aspects. First, the spatial–temporal variation characteristics of ESV, impact of land use/cover on ESV, and combination of spatial regression and geographic detector methods to analyze the driving factors of ESV spatial–temporal variation. For example, Hu et al. used InVEST model to explore the response of ESV to land use/cover change in the Pearl River Basin^12^; Huang et al. studied the spatial distribution of ESVs in the Lhasa River Basin^13^; Song et al. studied the Northeast Regional wetland ESV changes, identified the driving factors of ESV changes, and clarified the different driving factors’ contribution to ESV changes^14^. The second is using ESV as a criterion for establishing ecological compensation standards. For example, Wu et al. studied the Changtian Basin in Xixiu District, Anshun City and calculated the ESV, and compensation standards for cultivated land, forests, water areas, and orchards. Using carbon fixation and oxygen release and the InVEST model^15^, Tian et al. calculated the total ESV based on meteorological data, remote sensing data, and socio-economic data of the Chishui River in 2000, 2010, and 2015, and determined the compensation standards and priority compensation level in different areas of the basin^16^. The third step is to use FLUS, CA-Markov, and other models to forecast and simulate future land use changes, as well as estimate the profit and loss of ecosystem service value under various scenarios, in order to optimize land use layout. Hu et al. for example, used the GM and FLUS models to simulate land use change in Anhui Province under an ecological optimization scenario. They also used the revised model to estimate the ESV of Anhui Province from 1995 to 2030^17^.

The YRB covers a large area and is marked by complex geomorphological units, diverse ecosystem types, and distinct regional climate differences.The YRB's ecological environment is fragile, and vulnerable to global climate change. This area is an ecological corridor with ecological functions such as water conservation, windbreak and sand fixation, and biodiversity protection connecting the Qinghai-Tibet Plateau, Loess Plateau, and North China Plain. The YRB plays an important role of ecologic barrier in maintaining regional ecological security in China. It is a representative area for studying ESV and its equilibrium characteristics because of its complex and diverse topography and climate, which provide natural advantageous conditions for a diverse range of ecosystems and land use types. What's more, in response to vegetation degradation in the upper reaches of the YRB and soil erosion in the middle reaches, the Chinese government has implemented large-scale ecological engineering measures, such as the ecological protection and construction projects of the Three Rivers Sources, the comprehensive management projects of mountains, rivers, forests, fields, lakes, and grasses, and the projects of returning farmland to forests and grasslands. The land use/cover types in this region have changed dramatically, resulting in changes in ecosystem services on a spatial and temporal scale. As a result, more research into the spatial–temporal evolution characteristics of the YRB's ESV from the past to the present is required. Therefore, this study was undertaken to evaluate the ESV of the corresponding year and its spatial–temporal change characteristics, identify the cold and hot spots of the ESV, as well as the transfer path and distance of barycenter, reveal the changing law of ESV in the YRB, and analyze the response of ESV to land land-use changes, to provide scientific reference for rational land use and environmental protection.

## Materials and methods

### Study area

**Figure 1:**
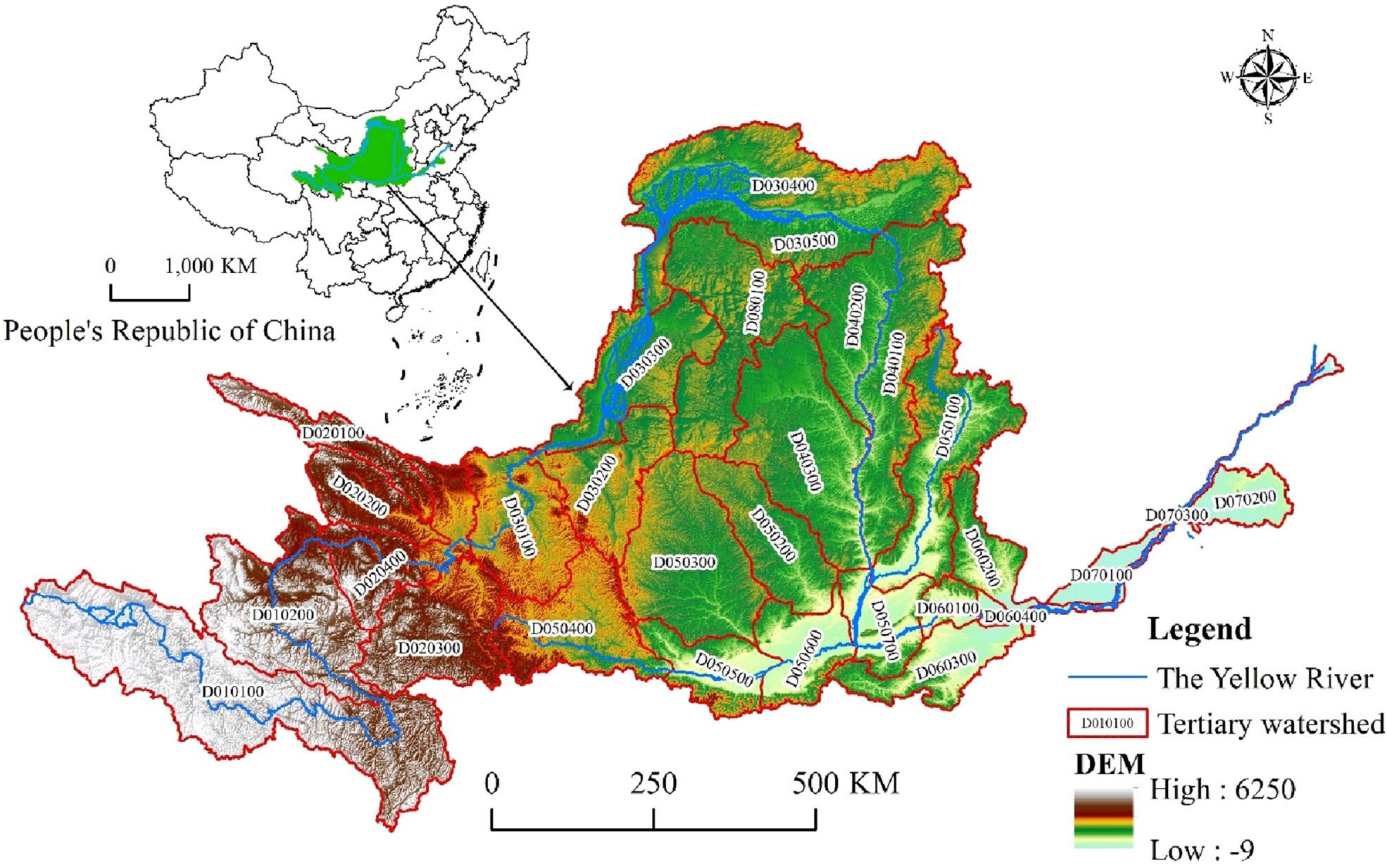
Scope of YRB and distribution of drainage system. http://www.geodata.cn/data/datadetails.html?dataguid=186217782730125&docid=19661http://www.geodata.cn/data/datadetails.html?dataguid=175123932227723&docid=18781.

The Yellow River originates from Yoguzonglie Basin, north of Bayan Har Mountains on the Qinghai-Tibet Plateau, and flows through Qinghai, Sichuan, Gansu, Ningxia, Inner Mongolia, Shanxi, Shaanxi, Henan, and Shandong, into the Bohai Sea in Kenli County, Shandong Province, with a total main stream length of 5464 km and 4480 m for the drop. The YRB is located between 96°–119°east longitude and 32°–42°north latitude (Fig. 1), with a length of about 1900 km from east to west, width of about 1100 km from north to south, and a drainage area of 79.5 × 10^4^ km^2^. The upper reaches of the Yellow River is located above Hekou Town, with a length of 3472 km and a drainage area of 42.8 × 10^4^ km^2^; while the middle reaches is from Hekou Town to Taohuayu, with a length of 1206 km and a drainage area of 34.4 × 10^4^ km^2^; the lower reaches is below Taohuayu, with a length of 786 km and a drainage area of only 2.3 × 10^4^ km^2^. The YRB has large territory with many mountains. The height difference between the east and the west is very significant. The topography of each region varies, as well as the climate in the basin. The YRB has a large seasonal difference, with annual precipitation ranging from 200 to 650 mm in most parts, and with more than 650 mm in the south of the middle-upper, and lower reaches, particularly the northern slope of the Qinling Mountains (700–1000 mm), as it gradually increases from northwest to southeast. Precipitation is unevenly distributed, with a north–south ratio greater than 5. The YRB can be divided into 8 secondary basins and 29 tertiary basins based on its water resources zoning (slices). The basin's resident population reached 152 million people in 2020, with a regional GDP of 9642.276 billion yuan and a 46.70% urbanization rate. The entire urbanization process is accelerating, and the conflict between development and the preservation of land space is becoming increasingly serious.

### Date source

The land use type data of YRB in 1990, 1995, 2000, 2005, 2010, 2015, and 2020 with a spatial resolution of 1 km was extracted from the Resource Science Data Center of the Chinese Academy of Sciences (http://www.resdc.cn/) remote sensing monitoring database of China's land use status. The primary data source for each phase is Landsat TM/ETM remote sensing images, which are interpreted and produced through human–computer interaction. Land use is divided into six categories by the land use change classification system: cultivated land, forest land, grassland, water area, construction land, and unused land.Because the negative effects of construction land on the YRB's ecosystem far outweigh the positive effects, its ESVs were omitted. The National Agricultural Product Cost and Benefit Data Compilation in 2020 and the Statistical Yearbook of 9 provinces, including Gansu, Qinghai, and Shandong, are used to compile statistical data such as grain output and grain prices.

### Evaluation methods of ESV

Ecosystem services can be classified into nine types, according to the findings of Constanza et al^5^. The "ecosystem equivalent table per unit area of ecosystems in China" proposed by Xie is the basis for this research^18^. Researchers can obtain the ESV coefficient (VC) table for each land land-use type in the YRB by correcting the YRB's socio-economic development status and the economic value of the annual natural grain yield per unit area of farmland. The specific correction process is as follows: the average yield per unit of grain is 4626.55 kg·ha^−1^ and the market price of grain is 0.35 USD·kg^−1^ in the YRB from 1990 to 2020. According to the calculation that the ecological service value per unit area of farmland is equal to 1/7 of the market economic value of average grain yield per unit qrea^14^, the equivalent factor of the ESV in the Yellow River Basin is 232.16USD·ha^−1^·a^−1^, and finally they obtain the ESV coefficient of the YRB, which is showed in Table 1.

**Table 1:** ESV coefficients per unit area of land use categories in YRB/(USD ha^−1^ a^−1^).

	Forest	Grassland	Cultivated land	Water	Unuselan
Food production	812.55	185.73	116.08	0.00	0.00
Raw materials	626.82	208.94	206.62	106.79	0.00
Water conservation	742.90	185.73	139.29	4731.35	6.96
Soil conservation	905.41	452.71	338.95	2.32	4.64
Waste disposal	304.13	304.13	380.74	4220.61	2.32
Gas regulation	756.83	253.05	164.83	578.07	78.93
Climate regulation	23.22	69.65	232.16	23.22	2.32
Biodiversity conservation	603.61	11.61	23.22	2.32	0.00
Entertainment	297.16	9.29	2.32	1007.56	2.32
Total	5072.62	1680.81	1604.20	10,672.25	97.51

Finally, the ESV of the study area is calculated as follows:

$${\text{ESV}} = \sum {\text{S}}_{{\text{k}}} \times {\text{VC}}_{{\text{k}}}$$

where ESV represents the value of ecosystem services (USD), S_k_ represents the area (hectare) of the kth land use type, and VC_k_ represents the ESV coefficient per unit area of the ecosystem type k (USD/ha).

### Local spatial autocorrelation of ESV

The degree of spatial correlation of a geographic thing or phenomenon at different locations in a given region expressed using spatial autocorrelation analysis (Moran's I)^19^, can be used to determine whether the distribution of ESV has agglomeration. Hot Spot Analysis (Getis-Ord Gi*) is used to measure the aggregation and differentiation characteristics of spatial changes in ESV (ESV), and to explore whether the spatial changes of ESV have high-value agglomeration (hot spot) and low-value agglomeration (cold spots) phenomenon^20^. It is possible to determine the location where the ESV high-value area or low-value area is clustered in space using hot spot analysis^21^. This was calculated using the formula:

$$\begin{gathered} {\text{Morans}}\;{\text{I}} = \frac{{{\text{n}}\sum\nolimits_{{{\text{j}} = 1}}^{{\text{n}}} {{\text{w}}_{{{\text{ij}}}} \left( {{\text{x}}_{{\text{i}}} - {\overline{\text{x}}}} \right)\left( {{\text{x}}_{{\text{j}}} - {\overline{\text{x}}}} \right)} }}{{\sum\nolimits_{{{\text{i}} = 1}}^{{\text{n}}} {\left( {{\text{x}}_{i} - {\overline{\text{x}}}} \right)^{2} \left( {\sum\nolimits_{{\text{i}}} {\sum\nolimits_{{\text{j}}} {{\text{w}}_{{{\text{ij}}}} } } } \right)} }} \hfill \\ Z\left( {G_{{\text{i}}}^{*} } \right){ = }\frac{{n\sum\nolimits_{j = 1}^{n} {w_{i,j}^{{}} x_{j} - X\sum\nolimits_{j = 1}^{n} {w_{i,j} } } }}{{\sqrt[{\text{s}}]{{\frac{{\left[ {{\text{n}}\sum\nolimits_{{{\text{j = }}1}}^{{\text{n}}} {{\text{w}}_{i,j}^{2} - \left( {\sum\nolimits_{j = 1}^{n} {w_{i,j} } } \right)^{2} } } \right]}}{{\left( {n - 1} \right)}}}}}} \hfill \\ X = \frac{1}{n}\sum\limits_{j = 1}^{n} {x_{i} } ,S{ = }\sqrt {\frac{1}{{\text{n}}}} \sum\limits_{{{\text{j = }}1}}^{N} {{\text{x}}_{{\text{j}}}^{2} } { - }\left( X \right)^{2} \hfill \\ \end{gathered}$$

where n is the number of spatial grid units in the study area, x_i_ and x_j_ are the observed values of spatial unit i and spatial unit j, respectively, and (${\text{x}}_{{\text{i}}} - {\overline{\text{x}}}$) is the deviation of the observed value from the mean value on the ith spatial unit, w_ij_ is the weight matrix of space units i and j.

### Land use change response resilience of ESV

The term "resilience" refers to how responsive one variable is to changes in another. This method was used to calculate the percentage change in ESV due to land use/land cover change in this paper^22^. The formula used was:

$$E = \left| {\frac{{\left( {ESV_{t1} - ESV_{to} } \right)/ESV_{t0} }}{LUP}} \right|,\quad LUP = \frac{{\mathop \sum \nolimits_{i = 1}^{n} \Delta L_{i} }}{{\mathop \sum \nolimits_{i = 1}^{n} L_{i} }}$$

where E is the elasticity index of ESV in response to land use change; t0 is the initial stage of the study, t1 is the end stage of the study; LUP is the percentage of land use change; ∆L_i_ represents the land use change area of i land use types, and Li represents the total area of i land use types.

### The barycenter shift of ESV

ESV's centre of gravity is based on the principles of population gravity centre and economic gravity centre, and it investigates ESV's temporal and spatial evolution from space. It also examines the ESV's barycenter and change trajectory in the YRB from four perspectives: barycentric coordinates, moving direction, distance, and administrative region location in 1990, 1995, 2000, 2005, 2015, and 2020. The following formula by Liu et al. was used for the calculation^23^:

$$X{ = }\frac{{\sum\nolimits_{{{\text{i = }}1}}^{{\text{n}}} {M_{{\text{i}}} X_{{\text{i}}} } }}{{\sum\nolimits_{{{\text{i = }}1}}^{{\text{n}}} {M_{{\text{i}}} } }},\quad Y{ = }\frac{{\sum\nolimits_{{{\text{i = }}1}}^{{\text{n}}} {M_{{\text{i}}} Y_{{\text{i}}} } }}{{\sum\nolimits_{{{\text{i = }}1}}^{{\text{n}}} {M_{{\text{i}}} } }}$$

Among them, M_i_ is the attribute value of the ESV of the region in this study; X_i_ and Y_i_ represent the geographic coordinates, respectively, and X and Y represent the barycentric coordinates of the ESV. The barycenter area in 1990, 1995, 2000, 2005, 2015, and 2020 was calculated using the formula:

$${\text{d = }}\rho \sqrt {\left( {{\text{x}}_{{{\text{i + t}}}} - {\text{ x}}_{{\text{i}}} } \right)^{2} + \left( {{\text{y}}_{{{\text{i + t}}}} {\text{ }} - {\text{ y}}_{{\text{i}}} } \right)^{2} }$$

where d represents the moving distance of the barycenter; (x_i_, y_i_) and (x_i+t_, y_i+t_) are the coordinates of the barycenter of the ESV in the ith and i + t-th years, respectively; and $\rho$ is the conversion rate between the plane coordinates and the geographic coordinates. Generally, researchers use a constant of 111.11 km.

## Results

### Analysis of changes in ecosystem services value in the YRB

The results showed that from 1990 to 2020, the total ecosystem services value in the YRB showed a dynamic trend of decrease-increase–decrease, with overall increasing trend, and a total increase of 31.85 × 10^10^ USD, with an average annual increase of 1.14 × 10^10^ USD (Table 2). This changing trend is consistent with land use cover change in the area. In 30a, YRB cultivated land decreased by 8663 km^2^, due to rapid urbanization. In addition, after year 2000, China began to implement the policy of returning farmland to forest and grassland on a large scale, which accelerated the reduction of cultivated land. Results again showed that the forest area increased by 30,933,093 km^2^, indicating that the implementation of “returning farmland to forest and grassland”policy achieved great results, thus increased the value of ecosystem services generated by forest land by 167.66 × 10^10^ USD. Grassland increased by 738 km^2^, as corresponding ESV increased by 28.73 × 10^10^ USD, while unused land decreased by 8131 km^2^, with 9.52 × 10^10^ USD ESV decrease. In general, the ecological protection and management measures in the YRB have achieved remarkable results, and ecosystem service values has been significantly improved due to forest and grassland increase.

**Table 2:** The value of ecosystem services in the YRB from 1990 to 2020.

Land use types	ESV(10^10^ USD)							
	1990	1995	2000	2005	2010	2015	2020	1990–2020
Cultivated land	3738.89	3740.72	3740.72	3738.89	3660.55	3644.44	3586.73	− 152.16
Forest	5833.27	5534.97	5788.60	5833.27	5997.59	5986.55	6000.93	167.66
Grass land	7131.05	7319.22	7151.14	7131.05	7162.28	7151.32	7159.78	28.73
Water	1646.70	1488.80	1595.52	1646.70	1602.87	1615.91	1643.84	− 2.86
Unused land	75.73	71.60	75.73	75.73	68.07	67.75	66.21	− 9.52
Total	18,425.65	18,155.32	18,351.72	18,425.65	18,491.37	18,465.96	18,457.50	31.85

In terms of ecosystem service structure in the YRB (Table 3), the relative proportions of various ESVs did not change significantly, resulting in relatively stable ESV structure. Soil conservation and waste disposal are the most important among them, accounting for about 37% of ESV's total value. The YRB ecosystem, as can be seen, emphasises the importance of soil conservation and waste disposal in the basin, with Climate regulation, Biodiversity conservation, and Entertainment accounting for only 11.99 percent of the total. Various services have changed to varying degrees during the study period. Waste disposal and climate regulation, for example, have suffered losses of 22.23 × 10^10^ USD and 20.29 × 10^10^ USD, respectively.The rest of the services showed an upward trend, among which the value of the Food production service increased the most, which was 19.03 × 10^10^ USD, owing to the obvious increase of the forest land and grassland area in the YRB.

**Table 3:** The value of individual ecosystem functions in the YRB from 1990 to 2020.

Service category	ESV(10^10^ USD)							
	1990	1995	2000	2005	2010	2015	2020	1990–2020
Food production	1992.89	1966.04	1991.24	1992.89	2017.00	2012.85	2011.92	19.03
Raw materials	2105.32	2090.50	2104.73	2105.32	2118.97	2114.30	2109.98	4.66
Water conservation	2702.36	2609.32	2671.39	2702.36	2703.09	2704.62	2714.93	12.57
Soil conservation	3755.78	3753.38	3753.91	3755.78	3776.60	3768.25	3760.84	5.06
Waste disposal	3180.43	3134.48	3156.27	3180.43	3159.82	3158.50	3158.20	− 22.23
Gas regulation	2478.59	2450.71	2472.27	2478.59	2491.18	2486.67	2484.43	5.84
Climate regulation	868.65	874.91	2478.59	868.65	859.08	856.27	848.36	− 20.29
Biodiversity conservation	797.83	763.63	797.56	797.83	816.46	814.84	815.78	17.95
Entertainment	543.79	512.36	537.03	543.80	549.16	549.65	553.06	9.27

### Spatial distribution and variation characteristics of ecosystem services in the YRB

**Figure 2:**
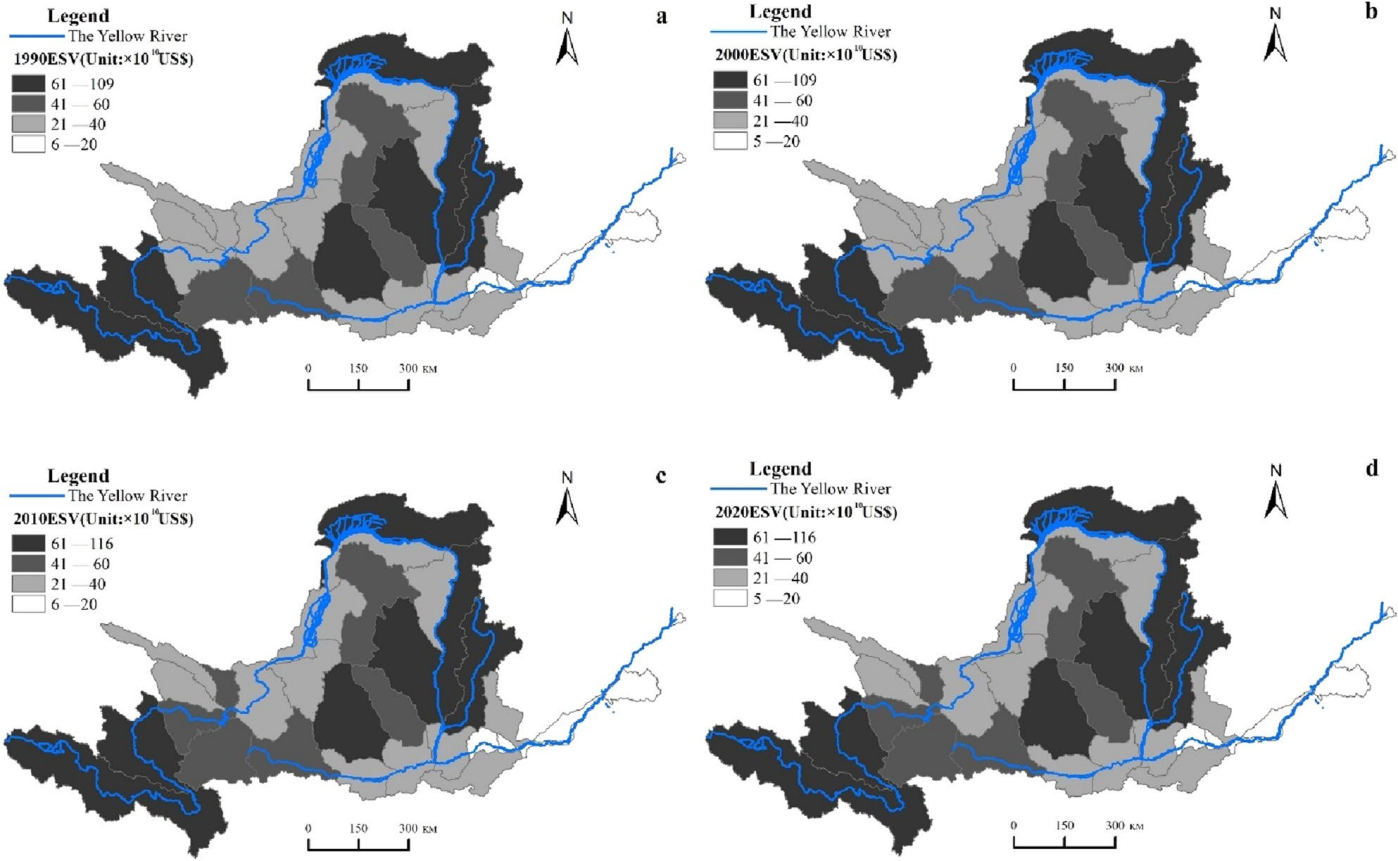
Spatial distribution of ESV changes in YRB from 1990 to 2020. (**a**) 1990, (**b**) 2000, (**c**) 2010, (**d**) 2020.

The total ESV value of the study area and changes in the value of each service could not reflect their spatial differences. To describe the temporal and spatial distribution pattern of ESV in the study area, the natural breakpoint method was used. This method was further used to classify ESV with reference to existing studies, and divided the area into four levels: low-value, lower-value, higher-value, and high-value areas. Takin the three-level watershed of the YRB as the statistical unit for analysis, the result showed that the higher the level, the higher the ESV. As shown in Fig. 2, from 1990 to 2020, the spatial characteristics of ESV were relatively stable. The YRB's upper reaches, from Shizuishan to the north bank of Hekou Town, the Fenhe River Basin, from Hekou Town to Longmen, and the Jinghe River Basin are all rich in high-values. The forest and grassland are relatively concentrated in the above-mentioned areas, the ESV coefficient is high, and the watershed area is large, resulting in a high total ESV. The higher value areas are mainly distributed in the areas from Longyang Gorge to Lanzhou main stream basin, the Daxia River and Tao River basin, and the Wei River basin. The area above Baoji Gorge and the inflow area fall in the transition zone between the high-value area and the lower-value area. For example, the transition area between the Loess and Qinghai-Tibet Plateaus is a higher-value area. The lower-value area mainly includes the Huangshui River Basin, the Datong River Basin, the basin below Lanzhou, and the Guanzhong Plain area. Thus, the unused land in this area is widely distributed. Due to the large area of construction land in the Guanzhong Plain, the ecosystem service value has shrunk. The low-value area is found in the YRB's lower reaches, which contains the most extensive and large area of construction land in the basin, has a poor ecosystem service function, and is also the YRB's most economically developed area. In terms of changes in the value of watershed ecosystem services, the number of watersheds at the ESV level did not change significantly between 1990 and 2020. The average ESV of the watershed is 40.52 × 10^10^ USD. There were 7 high-value, 5 higher-value, 12 lower-value, and 5 low-value watersheds respectively.

**Figure 3:**
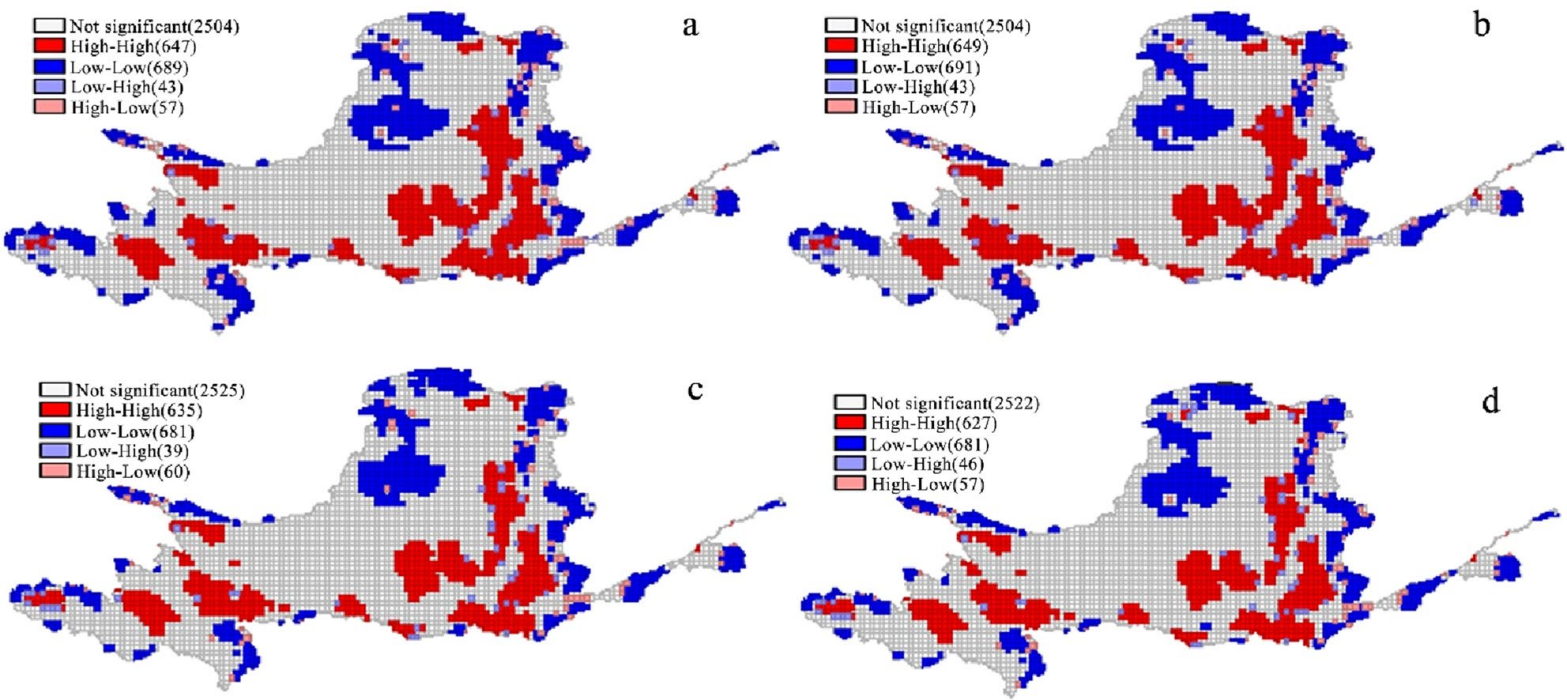
Spatial agglomeration characteristics of ESV in the YRB from 1990 to 2020. (**a**) 1990, (**b**) 2000, (**c**) 2010, (**d**) 2020.

The hotspot analysis revealed the spatial agglomeration characteristics and ESV evolution law in the YRB from 1990 to 2020 (Fig. 3). In most of the YRB, the ESV accumulation characteristics were not significant in space, and significant areas were dominated with high and low ESV accumulation. The Maqu-Longyangxia River Basin, Daxia River and Tao River Basin, the Datong River Basin, and Fen River Basin were the five core areas where ESV had the highest value. The Inner River, YRB's northern and eastern margins, and the lower reaches are primarily low-value agglomeration areas. The high-value agglomeration area and low-value agglomeration area did not change significantly in space from 1990 to 2020, but the number of grids in each decreased from 647 to 627 and 699 to 681, respectively. In general, the YRB's high-value agglomeration areas are strewn about, whereas the low-value agglomeration areas are scattered.

**Figure 4:**
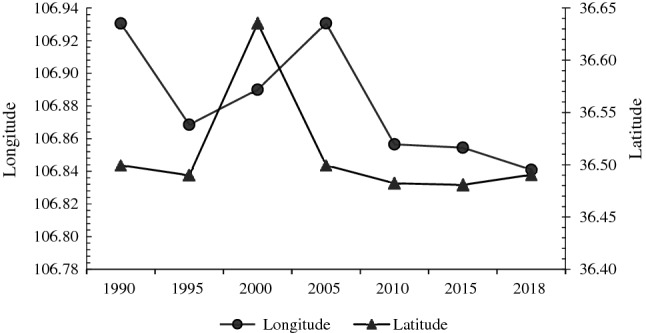
Barycenter coordinates of ecosystem services in the YRB from 1990 to 2020.

From 1990 to 2020, the barycenter coordinates of the ESV in the YRB remained stable between 106.78°–106.94° E and 36.40°–36.65° N (Fig. 4). During the study period, the ESV barycenter coordinates showed a transfer trajectory of first to southwest, then to northeast, and then to southwest. From the perspective of overall transfer direction, ESV barycenter shifted from northeast of Huanxian County to southwest from 1990 to 2020. The ESV in the northeast decreased, while that in southeast increased. From 1995 to 2000 and from 2000 to 2005, the migration distance of ESV barycenter in the YRB was longer by 16.33 km and 15.75 km, respectively, while the barycenter migration distance of ESV from 2005 to 2020 was shorter.

### Response of ecosystem services to land-use change

The area of land use type change in the YRB increased by 64,356 km^2^ between 1990 and 2020. Each land type's area has changed to varying degrees. Cultivated land and construction land are the two land types that have seen the most changes. The area of cultivated land has shrunk by 8663 km^2^, while the area of construction land has grown by 13,109 km^2^. In comparison to water, forest, and grassland, unused land has undergone significant transformations. However, in comparison to 1990, it shrunk by 8131 km^2^ in 2020. The forest increased by 3093 km^2^ while grassland increased by 738 km^2^. Ecosystem services are significantly impacted by changes in land use types. Using the spatial analysis method, the researcher introduces a resilience index to reflect ESV's response to land-use change in this paper. During 1990–2000 and 2000–2010, average elasticity of ESV change in the YRB relative to land use change was 0.27 and 0.44, respectively, but dropped to 0.04 during 2010–2020. This indicates that the disturbance capacity of land-use change on ecosystem services was low between 1990 and 2000, but increased between 2000 and 2010. Land-use change has had less of an impact on ecosystem services since 2010. The range of changes in land land-use types was wide during this time, but the average elasticity index was low because there were so many different types of land land-use changes, such as the conversion of forest land and cultivated land to construction land, and the conversion of forest land and water area to cultivated land. The decrease in ESV caused by the change in land use per unit area was minor. Furthermore, the forest land and grassland in the river basin have been effectively increased, as ESV has increased. Overall, the value of ecosystem services has remained relatively constant.

**Figure 5:**
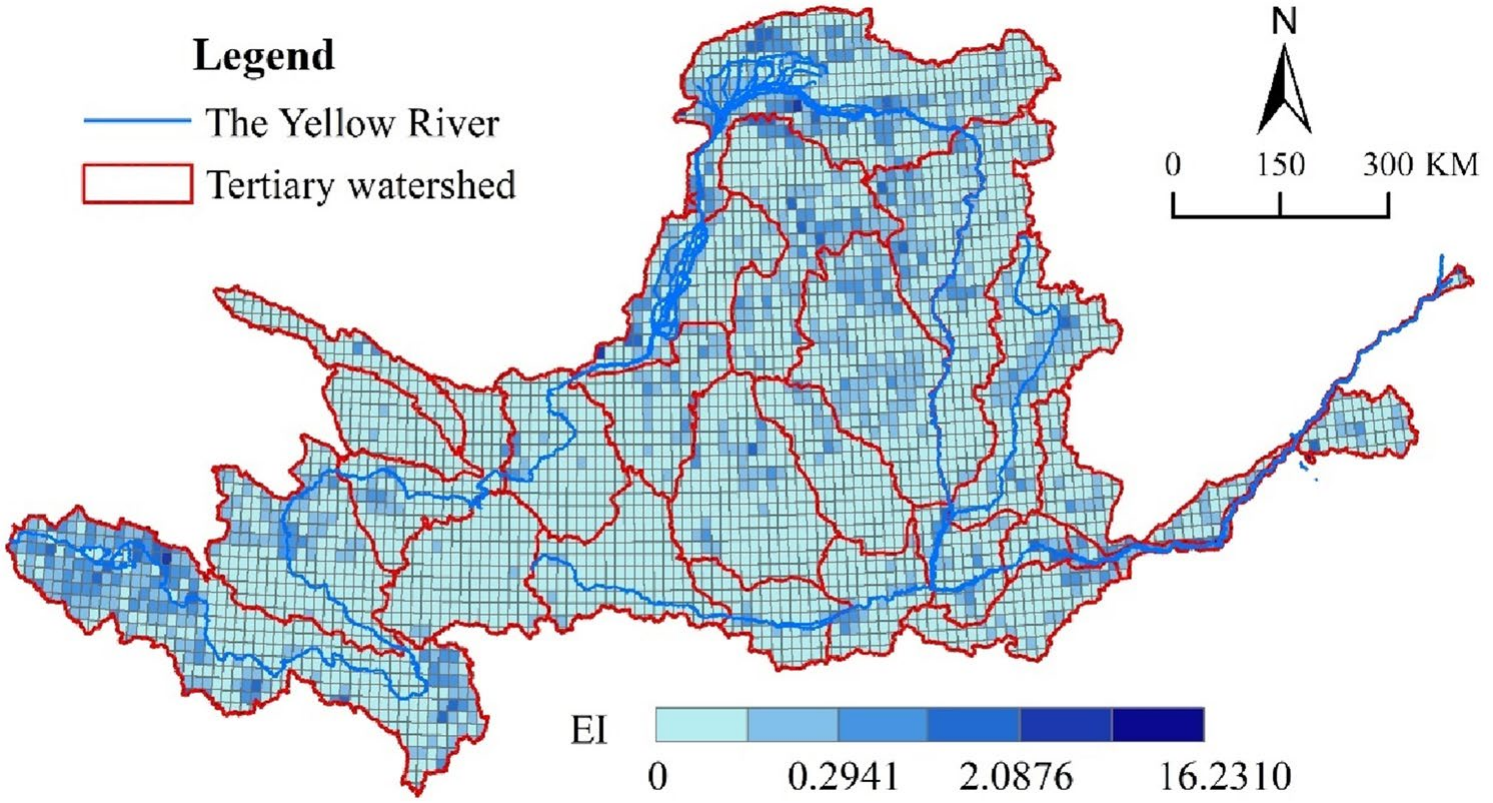
Spatial distribution of elastic coefficients in the YRB from 1990 to 2020.

Accurate spatial statistics on the elasticity index from 1990 to 2020 was carried out (Fig. 5). The elastic index of the upper YRB and Loess Plateau is higher, and the impact of land use change on ecosystem services is more apparent in this region, according to the findings. This is mainly due to the implementation of large-scale ecological engineering measures in response to vegetation degradation in the upper reaches of the YRB and soil erosion in the middle reaches (Loess Plateau), by the Chinese government. In addition, Lanzhou New District, Guanzhong Plain, and the lower Yellow River region also showed higher elasticity index. The above-mentioned regional development and construction, as well as human activities, have resulted in a rapid increase in construction land, resulting in a significant decline in ecosystem services and a higher resilience index as a result of rapid urbanisation.

In general, the land use types in the YRB have changed dramatically, and land type conversion is very common. The conversion of ecological land to urban construction land, as well as the conversion of unused and cultivated land to ecological land, has resulted in significant changes in ecosystem service value. This demonstrates that the basin's ecological construction projects have yielded positive environmental results.

## Discussion

Different scholars use different evaluation index systems and calculation methods to calculate the value of ecosystem services, according to literature reports. Yin et al. found that the ESV in the YRB showed a dynamic change from 1990 to 2020, first increasing and then decreasing, with an overall increasing trend, which is consistent with the trend of this study^24^. However, the accounting methods differ due to the different evaluation index systems chosen, resulting in numerical differences. The ESV of the YRB is calculated using the unit area value equivalent factor method in this study. This method has a relatively simple and comprehensive evaluation system, and it has proven to be a more effective method than evaluating benefits for evaluating ecosystem services value in larger areas. The grain yield and price in each county were not used in the calculation of the equivalent factor due to lack of data^25^. The accuracy and adaptability of ESV must be ensured during the evaluation process. More time-sensitive and local ecological indicators, as well as high-precision remote sensing data, should be incorporated into the evaluation system, as the relationship between humans and the natural environment continues to evolve; considering the locality of ESV research, more time-sensitive and local ecological indicators and high-precision remote sensing data should be incorporated into the evaluation system; the ESV evaluation model can fully consider ecological processes and will become the future research direction. Some scholars have found that the reduction of farmland and forest area can lead to ESV decline^26^. Due to the fact that urban construction land tends to occupy high-quality farmland, forest land, and grassland, the loss of ESV will result from the reduction of these land types. ESV decreased from 1990 to 1995 as forest land and water area decreased. ESV increased steadily from 1995 to 2010, owing primarily to increases in forest land and water area. ESV decreased from 2010 to 2020 as a result of policies such as "returning farmland to forests," "natural forest protection plan," and "Three North Protection Plan"^27^. Following 2010, as social and economic levels improved and urban development accelerated, construction land for residences, industrial parks, and public service facilities increased significantly, while grassland and cultivated land decreased, resulting in a decrease in ESV. The findings of the study agree with those of ^28^.

The hot and cold spot analysis method was used to characterise the spatial difference of ESV in this study. Many researchers have used it to investigate the spatial distribution of ESV^29^. Because the economy of upstream areas like Qinghai, Lanzhou, and Sichuan lags behind that of downstream areas, there is less construction land, grasslands are more widely distributed, and these areas have higher altitudes and lower accessibility to human activities. The year-round water supply provided by melting snow and ice nourishes the local grassland and primarily performs ecological service functions such as soil and water conservation^30,31^. The grassland forest resources of the northern Qilian Mountains, Taihang Mountains, and Qinling Mountains are abundant and hotspots like mediation, habitat, and human services are concentrated, and designated as ecological protection barriers. This ensure regional ecological security, define YRB's ecological corridor protection areas, create a system of nature reserves, and fully utilise the regulating role of ecological barriers. Cold spots, such as the Mu Us Sandy Land and Loess Plateau, are mostly found in YRB's middle and lower reaches. These areas are crisscrossed by ravines, hills, and beams, and have a fragile ecological environment, low forest and grass coverage, severe soil erosion, mostly unused and cultivated land, and weak ecosystem services, resulting in low ESV. There was flat terrain, convenient transportation, developed economy, and a lot of construction lands in downstream provinces like Henan and Shandong, resulting in ESV loss, which is represented by reduced biodiversity, fragmented habitat distribution due to land use changes, and species on the verge of extinction^32^. The fragmentation of habitat distribution caused by land use change puts species at risk of extinction. In this regard, the low-yield and poor-quality arable lands in the region are included in the new round of the project of returning farmland to forest and grassland (sand control and afforestation, planting grass, and eliminating desertification). Simultaneously, the gravity model was used to investigate the YRB's changing trend of ESV's "centre of gravity." The temporal and spatial changes in ESV, as well as changes in ESV over time, were analyzed for ecological impact and government decision-making. This is helpful in identifying problems and providing support for the formulation of ecological and environmental protection policies.

The results of this study revealed that water area contributed the most to total ESV. However, in recent years, due to accelerated urbanization, sharp increase in water consumption, discharge of industrial sewage, and the use of chemical fertilizers and pesticides, water pollution has been severely aggravated. Despite government's creation of "Transboundary Water Body Joint Protection Action" to address the shortcomings of the environmental protection law, penalties for illegal sewage discharge remain insufficient, and water pollution remains a serious problem. Therefore, the state's penalty for violations should be increased. Between 1990 and 2020, total ESV in YRB increased, owing largely to policies implemented by the national government, such as the conversion of farmland to forest and farmland to grassland. These policies made significant contributions to the promotion of ESV in YRB and security of regional ecological assets, as well as improving regional ecosystem, thus ensuring long-term ecosystem services benefits. Simultaneously, frequent human activity and expansion of urban construction land, renders large portions of cultivated lands occupied. As a result, cultivated land not only serves as an ecologically returning farmland and a protector of the environment, but also makes significant sacrifices in the name of social and economic development.As a result, maximising the safety of cultivated land remains critical in the process of regional land development and utilisation. Several suggestions were made in response to this: first, strengthen protection of basic farmland, check the borders of cultivated land, and balance cultivated and uncultivated lands; second, ESV should be included in decision-making when formulating regional land use planning policies; third, efficient management of returning farmland to forest and grassland project. Fourth, planning construction lands, avoiding disorderly development of construction lands, evaluating economic and intensive use of construction lands, digest and revitalise stock land, and improve the efficiency of urban land use in land use planning. Furthermore, because the upper, middle, and lower reaches of YRB are the ecological, energy, and economic centres^33^, the proposal of ecological protection countermeasures in YRB must classify the river basin according to the different protection priorities of the region and adapt measures to local conditions in order to strive for ecologically balanced development of human–environment relationships.

## Conclusion

This study used the equivalent factor method to evaluate the ESV of the YRB, revealing the numerical changes and spatial distribution characteristics of the ESV in the YRB, and delving into the ESV's response to changes in land use types. The following are the conclusions drawn:

1. In terms of time, from 1990 to 2020, the ESV in YRB showed an overall increasing trend, with total increase of 31.85 × 10^10^ USD, and average annual increase of 1.14 × 10^10^ USD. The ecosystem service value increased by 167.66 × 10^10^ USD and 28.73 × 10^10^ USD; In space, from 1990 to 2020, the spatial characteristics of ESV were relatively stable. The high-value areas were mainly distributed in the upper reaches of the YRB, from Shizuishan to the north bank of Hekou Town, the Fenhe River Basin, Hekou Town to Longmen, and the Jinghe River Basin; the low-value areas were distributed in the lower reaches of the YRB. The ESV of the YRB has the characteristics of spatial agglomeration in space. The barycenter coordinates of the ESV show a transfer trajectory of first southwest, then northeast, and then southwest.
2. From 2000 to 2010, the land-use change in the YRB had a greater impact on ESV, showing a high elasticity coefficient. Since 2010, the disturbance of ecosystem services caused by land-use change has become smaller. Spatially, the upper reaches of the YRB and the Loess Plateau have higher elastic indices, and the impact of land-use change on ecosystem services is more obvious, mainly because the large-scale implementation of ecological construction projects has significantly improved the ecological functions of the region.

## Data Availability

All data generated or analyzed during this study are included in this published article.
